# Abundant and cosmopolitan lineage of cyanopodoviruses lacking a DNA polymerase gene

**DOI:** 10.1038/s41396-022-01340-6

**Published:** 2022-11-10

**Authors:** Lanlan Cai, Yue Chen, Shiwei Xiao, Riyue Liu, Maoqiu He, Rui Zhang, Qinglu Zeng

**Affiliations:** 1 Department of Ocean Science, The Hong Kong University of Science and Technology, Clear Water Bay, Hong Kong, China; 2 Southern Marine Science and Engineering Guangdong Laboratory (Zhuhai), Zhuhai, China; 3 Southern Marine Science and Engineering Guangdong Laboratory (Guangzhou), Guangzhou, China; 4 Xiamen Cardiovascular Hospital, Xiamen University, Xiamen, China; 5 Fishery College, Zhejiang Ocean University, Zhoushan, 316022 China; 6 State Key Laboratory of Marine Environmental Science, College of Ocean and Earth Sciences, Xiamen University (Xiang’an), Fujian, China; 7 HKUST Shenzhen-Hong Kong Collaborative Innovation Research Institute, Futian, Shenzhen, China

**Keywords:** Virus-host interactions, Microbial ecology

## Abstract

Cyanopodoviruses affect the mortality and population dynamics of the unicellular picocyanobacteria *Prochlorococcus* and *Synechococcus*, the dominant primary producers in the oceans. Known cyanopodoviruses all contain the DNA polymerase gene (DNA *pol*) that is important for phage DNA replication and widely used in field quantification and diversity studies. However, we isolated 18 cyanopodoviruses without identifiable DNA *pol*. They form a new MPP-C clade that was separated from the existing MPP-A, MPP-B, and P-RSP2 clades. The MPP-C phages have the smallest genomes (37.3–37.9 kb) among sequenced cyanophages, and show longer latent periods than the MPP-B phages. Metagenomic reads of both clades are highly abundant in surface waters, but the MPP-C phages show higher relative abundance in surface waters than in deeper waters, while MPP-B phages have higher relative abundance in deeper waters. Our study reveals that cyanophages with distinct genomic contents and infection kinetics can exhibit different depth profiles in the oceans.

## Introduction

Viruses are the most abundant biological entities in the oceans and play important roles in the marine ecosystem [1, 2]. Marine viruses can control microbial population dynamics through the lysis of host cells, mediate horizontal gene transfer among host genomes, and influence the marine biogeochemical cycle by expressing virus-encoded auxiliary metabolic genes during infection [3–7]. Due to the rapid developments in culture-independent approaches, especially viral metagenomics, our knowledge about the genetic diversity of marine viruses has increased considerably in recent years [8–10]. Despite a large number of newly discovered viral genomes by metagenomics, more than 90% of sequencing reads in many marine samples are derived from unknown viruses, indicating a need for culture-dependent efforts to further characterize viral dark matter [11].

One of the most important and widely distributed groups of marine viruses are cyanophages (viruses that infect cyanobacteria) [12–14]. Marine cyanobacteria, mainly of the genera *Prochlorococcus* and *Synechococcus*, are the most abundant photosynthetic organisms and responsible for more than 25% of primary production in the open ocean, contributing significantly to global CO_2_ fixation [15, 16]. In the ocean 1%–15% of marine cyanobacteria are infected by cyanophages [17, 18], and in some oceanic regions more than 46% of cyanobacteria can be killed by phages daily [19]. Via infection, cyanophages could greatly impact the population size, community dynamics [18, 20, 21], genome diversity and evolution of cyanobacteria [22, 23], as well as marine biogeochemical cycles [24–28]. Marine cyanophages isolated thus far include several distinct groups: cyanopodoviruses belonging to the T7-like group, cyanomyoviruses belonging to the T4-like or TIM5-like groups, and cyanosiphoviruses [29–35]. The T7-like cyanopodoviruses are frequently isolated and well represented in many oceanic metagenomic datasets, representing a ubiquitous and ecologically important viral component in the ocean [36–38].

The first sequenced marine cyanopodovirus genome (cyanophage P60 isolated from *Synechococcus* WH7805) contains the DNA polymerase gene (DNA *pol*) that is conserved in podoviruses infecting different heterotrophic bacteria [39, 40]. So far, DNA *pol* is present in all 18 published marine cyanopodovirus genomes [29, 34, 40–42] and 3 unpublished cyanopodovirus genomes in GenBank (Table 1). Based on the phylogenetic tree of DNA *pol*, marine cyanopodoviruses form a monophyletic MPP (Marine Picocyanobacteria Podovirus) subgroup that is further divided into MPP-A and MPP-B clusters [43], with the cyanophage P-RSP2 isolated from *Prochlorococcus* MIT9302 being an outlier [34].

**Table 1:** General features of the cyanopodovirus genomes from this study and from previous studies.

Phage	Clade	Original host	Isolation site	Depth	Genome (bp)	GC %	ORFs	Accession	Reference
P-SCSP1a	MPP-C	*Prochlorococcus* MED4	South China Sea	Surface	37,777	38.7	36	OM416763	This study
P-SCSP1b	MPP-C	*Prochlorococcus* MED4	South China Sea	Surface	37,821	38.8	40	OM416764	This study
P-SCSP1c	MPP-C	*Prochlorococcus* MED4	South China Sea	Surface	37,789	38.8	36	OM416765	This study
P-SCSP1d	MPP-C	*Prochlorococcus* MED4	South China Sea	Surface	37,752	38.8	36	OM416766	This study
P-SCSP1f	MPP-C	*Prochlorococcus* MED4	South China Sea	Surface	37,858	38.7	39	OM416767	This study
P-SCSP1g	MPP-C	*Prochlorococcus* MED4	South China Sea	Surface	37,811	38.7	40	OM416768	This study
P-SCSP1h	MPP-C	*Prochlorococcus* MED4	South China Sea	Surface	37,844	38.7	40	OM416769	This study
P-SCSP1i	MPP-C	*Prochlorococcus* MED4	South China Sea	Surface	37,812	38.7	40	OM416770	This study
P-SCSP1k	MPP-C	*Prochlorococcus* MED4	South China Sea	Surface	37,771	38.7	37	OM416771	This study
P-SCSP1l	MPP-C	*Prochlorococcus* MED4	South China Sea	Surface	37,821	38.7	40	OM416772	This study
P-SCSP1m	MPP-C	*Prochlorococcus* MED4	South China Sea	Surface	37,812	38.7	40	OM416773	This study
P-SCSP1n	MPP-C	*Prochlorococcus* MED4	South China Sea	Surface	37,812	38.7	40	OM416774	This study
P-SCSP1o	MPP-C	*Prochlorococcus* MED4	South China Sea	Surface	37,752	38.8	36	OM416775	This study
P-SCSP1p	MPP-C	*Prochlorococcus* MED4	South China Sea	Surface	37,844	38.7	39	OM416776	This study
P-SCSP1q	MPP-C	*Prochlorococcus* MED4	South China Sea	Surface	37,754	38.7	40	OM416777	This study
P-SCSP1s	MPP-C	*Prochlorococcus* MED4	South China Sea	Surface	37,849	38.7	39	OM416778	This study
P-SCSP1u	MPP-C	*Prochlorococcus* MED4	South China Sea	Surface	37,861	38.7	38	OM416779	This study
P-SCSP2	MPP-C	*Prochlorococcus* MED4	South China Sea	Surface	37,264	34.2	44	OM416780	This study
P-RSP2	P-RSP2	*Prochlorococcus* MIT9302	Red Sea	Surface	42,257	34.0	48	HQ332139	[34]
P60	MPP-A	*Synechococcus* WH7805	Satilla River Estuary	Surface	46,675	53.3	55	AF338467	[39]
Syn5	MPP-A	*Synechococcus* WH8019	Sargasso Sea	Surface	46,214	55.0	61	EF372997	[41]
S-CBP2	MPP-A	*Synechococcus* CB0208	Chesapeake Bay	Surface	46,237	55.0	53	KC310806	[40]
S-CBP42	MPP-A	*Synechococcus* WH7803	Chesapeake Bay	Surface	45,218	54.6	57	KC310805	[40]
P-SSP9	MPP-A	*Prochlorococcus* SS120	BATS	100 m	46,997	40.5	54	HQ316584	[34]
P-SSP7	MPP-B	*Prochlorococcus* MED4	Sargasso Sea	100 m	44,970	38.8	54	AY939843	[29]
P-SSP5	MPP-B	*Prochlorococcus* MIT9515	North Pacific gyre	12 m	47,055	39.2	55	GU071100	GenBank
P-RSP5	MPP-B	*Prochlorococcus* NATL1A	Red Sea	130 m	47,741	38.7	68	GU071102	[34]
P-HP1	MPP-B	*Prochlorococcus* NATL2A	HOT	25 m	47,536	39.9	66	GU071104	[34]
P-SSP2	MPP-B	*Prochlorococcus* MIT9312	BATS	120 m	45,890	37.9	59	GU071107	[34]
P-GSP1	MPP-B	*Prochlorococcus* MED4	Gulf Stream	80 m	44,945	39.6	53	HQ332140	[34]
P-SSP3	MPP-B	*Prochlorococcus* MIT9312	BATS	100 m	46,198	37.9	56	HQ332137	[34]
P-SSP10	MPP-B	*Prochlorococcus* NATL2A	BATS	100 m	47,325	39.2	52	HQ337022	[34]
P-SSP6	MPP-B	*Prochlorococcus* MIT9515	BATS	100 m	47,039	39.2	54	HQ634152	[34]
S-CBP1	MPP-B	*Synechococcus* CB0101	Baltimore Inner Harbor	Surface	46,547	47.6	51	KC310802	[40]
S-CBP3	MPP-B	*Synechococcus* CB0101	Chesapeake Bay	Surface	45,871	47.0	55	KC310803	[40]
S-CBP4	MPP-B	*Synechococcus* CB0101	Chesapeake Bay	Surface	44,147	44.4	49	KC310804	[40]
S-RIP1	MPP-B	*Synechococcus* WH8101	Narragansett Bay	Surface	44,892	42.9	54	HQ317388	GenBank
S-RIP2	MPP-B	*Synechococcus* WH7803	Rhode Island Sound	Surface	45,728	47.3	56	HQ317389	GenBank
S-SBP1	MPP-B	*Synechococcus* WH7803	Sanya Bay	Surface	45,519	46.8	55	MT424636	[42]

Given its conservation in podovirus genomes and crucial function in phage genome replication, DNA *pol* has been widely used as a genetic marker for quantification and genetic diversity studies of cyanopodoviruses [7, 36, 37, 44–46]. However, evidence indicating the existence of cyanopodoviruses without DNA *pol* is accumulating. For example, the work examining the depth distribution of cyanopodoviruses revealed significantly lower abundances based on DNA *pol* than those based on the terminase gene in some samples [7]. The discrepancy may be due to the inability to recover all naturally occurring cyanopodoviruses by DNA *pol*. Furthermore, a polony method based on T4-like and T7-like cyanophage primers showed that the ratio of cyanophages to the total virus-like particles in the North Pacific Subtropical Gyre is 2–3 fold lower than that of cyanobacteria to the total bacteria, suggesting unknown cyanophage groups [45]. Likewise, unknown cyanophage types were found in different oceanic regions, and they could not be amplified by PCR primers targeting lineage-specific signature genes for the T4-like (portal protein gene *g20*), TIM5-like (DNA *pol*), and T7-like (DNA *pol*) cyanophages [47, 48].

Here we report a novel lineage of cyanopodoviruses isolated from the South China Sea using *Prochlorococcus* MED4 as the host, and we named this lineage as the MPP-C cluster. The newly isolated MPP-C cyanopodoviruses did not encode DNA *pol* and auxiliary metabolic genes. They accounted for up to 91% of the cyanopodovirus reads in certain oceanic regions, substantially increasing the total number of hitherto known marine cyanopodoviruses in the global ocean. Furthermore, metagenomic analyses suggested that the MPP-C phages have higher relative abundance in surface ocean than in deeper ocean, which could be linked to their distinct genomic contents and infection kinetics. Our discovery of the overlooked cyanopodovirus group sheds light on the diversity of T7-like viruses in the world’s oceans.

## Results

### Isolation and genome sequencing of cyanopodoviruses without DNA *pol*

Using *Prochlorococcus* MED4 as the host, a high-light-adapted strain abundant in the oceans [49], we isolated 18 cyanopodoviruses from the surface water of the South China Sea, the largest marginal sea with *Prochlorococcus* as the dominant primary producer [50]. Of the 18 cyanopodoviruses, 17 isolates were highly similar, sharing 95.99% to 99.95% average nucleotide identity (ANI) between each other (Supplementary Figure 1). Since phages with genomic identity above 95% can be considered as the same population [8–10], these 17 isolates were grouped into the P-SCSP1 subgroup, with the first P standing for *Prochlorococcus*, SCS for South China Sea, and the second P for podovirus. The other isolate, P-SCSP2, showed 88.3%–88.4% ANI with the P-SCSP1 subgroup (Supplementary Figure 1, Supplementary Table 1).

**Fig. 1:**
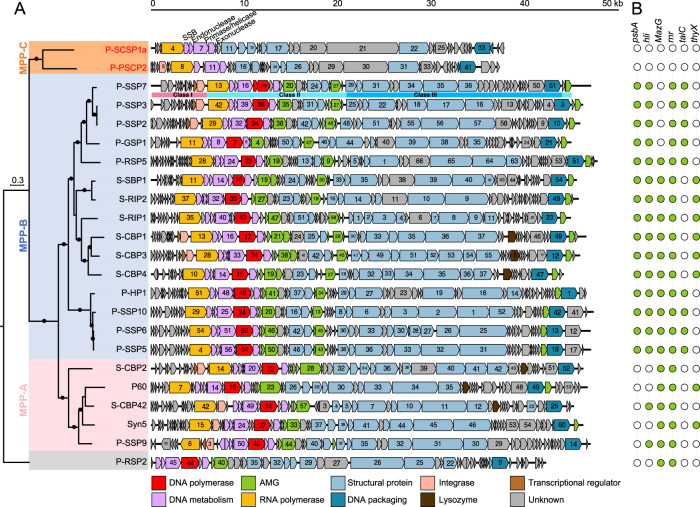
Comparison of cyanopodovirus genomes and their auxiliary metabolic genes. **A**. Cyanopodovirus genomes. The phylogenetic tree on the left was generated from the amino acid sequences of the concatenated nine core genes shared by the 39 cyanopodoviruses using a maximum likelihood method. A black dot in a branch indicated bootstrap values larger than 80%. The scale bar represented 0.3 fixed mutations per amino acid position. The names of cyanopodoviruses isolated in this study were shown in red, and those of other phages were shown in black. Cyanophages belonging to different clades were shaded by different colors: orange for MPP-C clade, blue for MPP-B, pink for MPP-A, and grey for P-RSP2. In the MPP-C clade, P-SCSP1a was shown as a representative of cyanophages P-SCSP1a to P-SCSP1u, which share highly similar genomic organizations. In each cyanophage genome, different colors indicated genes with different functions: DNA *pol* in red, other genes related to DNA metabolism in purple, auxiliary metabolic genes (AMGs) in green, RNA *pol* in light brown, structural genes in light blue, DNA packaging genes in dark blue, integrase gene in pink, lysozyme gene in dark brown, transcriptional regulator genes in light brown, and genes with unknown function in grey. Three classes of genes, which was shown to be sequentially transcribed in cyanophage P-SSP7, were indicated below the P-SSP7 genome. Abbreviation: SSB, single-stranded DNA binding protein. **B** Auxiliary metabolic genes in cyanopodovirus genomes. Green and white circles indicated the presence and absence of a gene, respectively. Gene abbreviations: *psbA*, photosystem II D1 protein; *hli*, high light inducible protein; *MazG*, pyrophosphatase; *rnr*, ribonucleotide reductase; *talC*, transaldolase; *thyX*, thymidylate synthase.

The genomes of these newly isolated cyanopodoviruses exhibited a well-conserved T7-like genomic architecture (Fig. 1A), in which three classes of genes were grouped from the left to the right and were shown to be transcribed sequentially in cyanopodoviruses P-SSP7 and S-SBP1 [42, 51]. It is thought that class I genes can modify the host transcriptional machinery, class II genes are mainly involved in DNA metabolism and replication, and class III genes are responsible for phage DNA maturation and virion assembly [51]. DNA *pol* is always located among class II genes in the 21 previously sequenced cyanopodoviruses (Fig. 1A). However, no DNA *pol* could be identified in the cyanopodoviruses we isolated from the South China Sea (Fig. 1A).

### Cyanopodoviruses without DNA *pol* form a new cyanophage MPP-C clade

To study the phylogenetic relationships of cyanopodoviruses with and without DNA *pol*, we analyzed the core genes shared by all marine cyanopodoviruses. The previously available 21 marine cyanopodoviruses (Table 1) contained 349 unique genes (the pan-genome) and 15 core genes (the core-genome) [40]. After adding the 18 cyanopodovirus genomes we sequenced in this study, the pan-genome of the 39 marine cyanopodoviruses increased to 379 genes (Supplementary Figure 2A) and the core-genome decreased to nine genes (Supplementary Figure 2B). Although the rate of increase seemed to slow down as more genomes were added, the gene accumulation curve was still far from saturated, suggesting undiscovered diversity within cyanopodovirus populations. Of the nine core genes, two encoded proteins involved in DNA metabolism and replication (exonuclease and DNA primase/helicase), five encoded phage structural proteins (major capsid protein, capsid assembly protein, portal protein, tail tube proteins A and B), and two encoded key components of DNA packaging machine (terminase large and small subunits, TerL and TerS, respectively) (Supplementary Figure 3).

The phylogenetic tree based on the concatenated nine core genes divided previously studied cyanopodovirus genomes [34] into the MPP-A and MPP-B clades, with cyanophage P-RSP2 as an outlier (Fig. 1A). The 18 newly isolated cyanopodoviruses without DNA *pol* formed a distinct clade, branching as a sister lineage to MPP-A and MPP-B clades, which we denoted as the MPP-C clade (Fig. 1A). Phylogenetic trees based on each of the nine core genes also supported the separation of the MPP-C clade from the MPP-A and MPP-B clades (Supplementary Figure 3).

The six genes absent from the previously defined core-genome [40] included two related to virion structural proteins (tail fiber and internal core protein), three involved in DNA metabolism and replication (DNA polymerase, ssDNA binding protein, and endonuclease), and one hypothetical gene. It should be noted that phages of the P-SCSP1 subgroup have genes encoding endonuclease (e.g., gene 06 of P-SCSP1a) that are orthologous to endonucleases of previously known cyanopodoviruses. However, no recognizable gene encoding endonuclease was found in P-SCSP2 (Fig. 1A), implying the divergency of gene content within the MPP-C clade. Gene 05 in P-SCSP1a, which is orthologous to gene 09 in P-SCSP2, is predicted to encode ssDNA binding protein since it shows 47.5% of similarity with ssDNA binding protein of Pelagibacter phage HTVC109P (P109_gp13) [52] via HMMER search (Fig. 1, Supplementary Table 1). However, they do not fall into the same orthologous group with ssDNA binding proteins of previously known cyanopodoviruses. Furthermore, we speculate that g20/g21 of P-SCSP1a and g29/g31 of P-SCSP2 may encode internal virion proteins based on their gene sizes and positions in the genomes. However, none of these four genes can be functionally annotated or show significant hits with genes of published phages. In addition, these four genes show no similarity between P-SCSP1a and P-SCSP2. During infection of coliphage T7, the internal core proteins are thought to form a trans-envelope channel that allows the injection of phage DNA into host cells to initiate infection [53]. The absence of these six genes from the core-genome suggests that the MPP-C phages may have unique replication mechanisms that are distinct from the MPP-A and MPP-B phages.

### MPP-C phages do not encode auxiliary metabolic genes and have small genomes

Cyanophages often encode host-like metabolic genes, termed auxiliary metabolic genes (AMGs), whose products were hypothesized to increase phage fitness under stress conditions [54–57]. Genes for photosystem II D1 protein (*psbA*), high light inducible protein (*hli*), pyrophosphohydrolase (*mazG*), ribonucleotide reductase (*rnr*), transaldolase (*talC*), and thymidylate synthase (*thyX*) have been found in cyanopodovirus genomes [40, 54, 58]. Out of these six AMGs, each of the previously studied cyanopodovirus genomes contained two to five (Fig. 1B) [29, 34, 40]. However, no recognizable AMGs were found in the genomes of MPP-C phages (Fig. 1), suggesting that the MPP-C phages have special metabolic mechanisms and evolutionary trajectories that are distinct from other cyanopodovirus clades.

Consistent with the lack of DNA *pol* and AMGs, cyanopodoviruses in the MPP-C clade have smaller genomes. Cyanophages in the MPP-A and MPP-B clades had genome sizes of 46.2 ± 0.6 kb and 46.1 ± 1.1 kb, respectively, and the outlier phage P-RSP2 had a genome size of 42.3 kb (Table 1). Cyanophages in the MPP-C clade had genome sizes from 37.3 kb to 37.9 kb (37.8 ± 0.1 kb), which were significantly smaller than those of MPP-A, MPP-B, and P-RSP2 phages (*p* < 0.001, t-test) (Table 1, Fig. 1). A bioinformatic analysis of double-stranded DNA viruses showed that viruses with small genomes tend not to encode their own DNA polymerase, especially for viral genomes smaller than 40 kb [59]. Our study provides further evidence for the possible correlation between DNA polymerase genes and viral genome sizes.

### Host range, morphology, and infection kinetics of MPP-C phages

The host ranges of MPP-C phages were tested against three marine *Synechococcus* strains (WH7803, WH8012, WH8102) and nine *Prochlorococcus* strains, of which six belong to the high-light-adapted ecotypes (MED4, AS9601, MIT9312, MIT9202, MIT9215, MIT9301) and three belong to the low-light-adapted ecotypes (MIT9313, NATL1A, NATL2A). We found that the MPP-C phages were highly host-specific and could only infect the original host *Prochlorococcus* MED4, which is consistent with previous studies showing very narrow host ranges for cyanopodoviruses [12, 34, 35, 43].

**Fig. 2:**
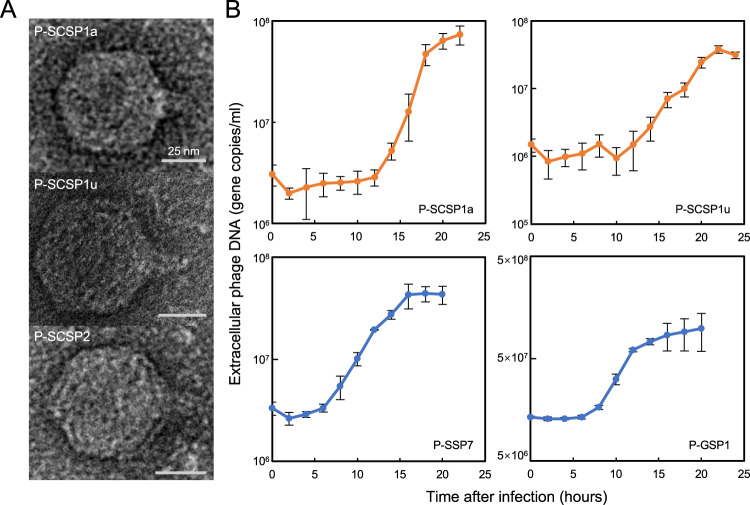
Transmission electron micrographs and infection kinetics of cyanopodoviruses. **A**. Transmission electron micrographic images of three representative phages belonging to the MPP-C clade. Scale bars represented 25 nm. **B**. Infection dynamics of MPP-C (top figures) and MPP-B (bottom figures) phages infecting *Prochlorococcus* MED4. The abundances of phage DNA in extracellular media were determined using qPCR. The average value and standard deviation of three biological replicates were shown.

Three MPP-C phages (P-SCSP1a, P-SCSP1u, and P-SCSP2) were selected for morphological characterization (Fig. 2A). The transmission electron micrographs of the three phage particles all showed icosahedral capsids and short tails (Fig. 2A), resembling the morphology of the podovirus T7. The capsid diameters of the three MPP-C phages were ~54 nm, slightly smaller than those of Syn5 (60 nm, MPP-A group) and P-SSP7 (65.5 nm, MPP-B group) [41, 60, 61].

To compare the infection kinetics of cyanopodoviruses under identical conditions, we used *Prochlorococcus* MED4 as the host, which can be infected by both the MPP-C and MPP-B phages. Two MPP-B phages P-SSP7 and P-GSP1 showed similar one step infection dynamics with latent periods of 6–8 h, which are consistent with previous studies of these two phages [51, 62, 63]. Following the latent period, the amounts of extracellular phage DNA for P-SSP7 and P-GSP1 increased exponentially until the plateaus were reached at ~16 hours after infection (Fig. 2B). Compared to MPP-B phages, the MPP-C phages P-SCSP1a and P-SCSP1u had longer latent periods of 12 h and 15 h, respectively, and the extracellular phage DNA reached the plateaus at ~22 h after infection (Fig. 2B). However, despite numerous attempts, we were unable to get a stable one step growth curve for P-SCSP2. The experiments yielded no burst or burst at different time points ranging from ten hours to a few days after inoculation (data not shown). The plateau phase, which represents the end of a single infection cycle, was hardly observed. The variations in lysis time of P-SCSP2 may be attributed to the presence of an integrase gene in its genome, which could facilitate transient integration of cyanophage genome into the host genome [64].

### The MPP-C clade is highly abundant in the global surface oceans

**Fig. 3:**
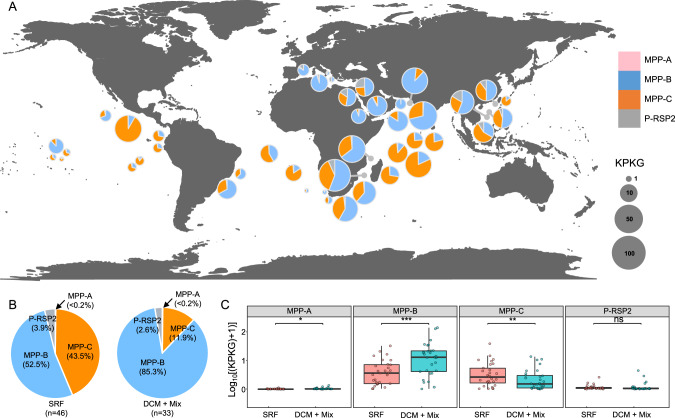
The global distribution of cyanopodovirus clades. **A**. Relative abundances of the four cyanopodovirus clades in surface waters. Cyanophages in the MPP-A, -B, and -C clades were indicated in pink, blue, and orange, respectively. P-RSP2–like cyanophages were indicated in grey. The size of a pie chart represented the overall recruitment frequency of the cyanopodovirus population in a sampling site, expressed as the total recruited nucleotides of reads (kb) per kb of viral genome per Gb of virome dataset (KPKG). **B**. The proportions of different cyanopodovirus clades in sampling sites from surface (SRF, 46 sites), deep chlorophyll maximum and the bottom of mixed layer when no deep chlorophyll maximum (DCM + Mix, 33 sites in total). **C**. Comparison of recruitment frequency of cyanopodovirus clades in the SRF and DCM + Mix sites. Analyses were performed for the 29 sites where both SRF and DCM/Mix waters were sampled. Recruitment frequency was displayed as log_10_(KPKG + 1). Paired t-tests were applied to compare SRF and DCM + Mix samples: * for *p* < 0.05, ** for *p* < 0.01, *** for *p* < 0.001, and ns for no significance.

To compare the global distributions of MPP-A, -B, -C, and P-RSP2 phages, we analyzed the viromic datasets of the *Tara* Oceans project covering different oceanic zones [8, 9]. We also analyzed viromes from surface waters of the South China Sea [65], where the MPP-C phages were isolated. The recruited numbers of cyanopodovirus reads were high in the surface waters of the Indian, Atlantic and Pacific Oceans, and the Mediterranean and Red Seas (Fig. 3A, Supplementary Table 2). Low numbers of cyanopodovirus reads were identified from the Arctic Ocean (Supplementary Table 2), which was reasonable given the low number of cyanobacteria in these regions [66].

In surface waters, the MPP-C phages were highly abundant, accounting for 43.5% of total cyanopodovirus reads across the global ocean, while the contributions of MPP-B and P-RSP2 clades were 52.5% and 3.9%, respectively (Fig. 3B). In the deep chlorophyll maximum (DCM) and the bottom of mixed layer when there was no deep chlorophyll maximum (Mix), the MPP-B clade was dominant (85.3%), followed by MPP-C (11.9%) and P-RSP2 (2.6%) clades (Fig. 3B). In the surface, DCM, and Mix samples, the MPP-A clade was always less than 0.2% of cyanopodovirus reads (Fig. 3B). Globally, the MPP-C phages had higher relative abundance in surface waters than in DCM and Mix waters, while MPP-B phages showed higher relative abundance in DCM and Mix waters than in surface waters (Fig. 3C). P-RSP2–like phages had similar relative abundances in both depths (Fig. 3C).

**Fig. 4:**
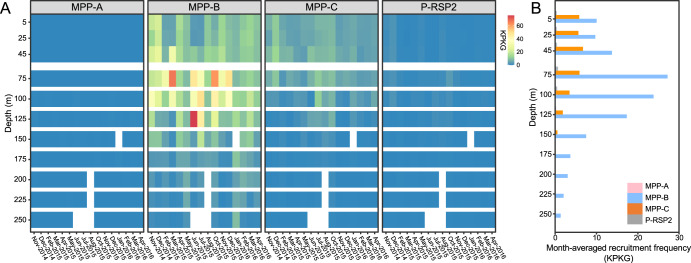
The spatiotemporal distribution of different cyanopodovirus clades in the North Pacific Subtropical Gyre. Data were recruited from the ALOHA 2.0 viromes covering 0.02–0.2 μm virus-enriched samples across 1.5 years at 12 depths [67]. **A** Temporal variability in recruitment frequency of different cyanopodovirus clades. Color bar showed the normalized recruitment which was expressed as the total recruited nucleotides of reads (kb) per kb of viral genome per Gb of virome dataset (KPKG). White shading indicated data unavailable from the ALOHA 2.0 viromes. **B** Depth profiles of month-averaged recruitment frequency of cyanopodoviruses. MPP-A, pink; MPP-B, blue; MPP-C, orange; P-RSP2–like cyanophages in grey.

To further compare the depth profiles of the four cyanopodovirus clades at different months of a year, we analyzed the ALOHA 2.0 viral dataset [67]. In this dataset, a total of 188 virus metagenomes were generated from seawater samples across 1.5 years at 12 depths (5–500 m) in the North Pacific Subtropical Gyre, enabling us to examine the spatiotemporal dynamics of viruses at a high resolution. Consistent with the global patterns (Fig. 3C), the MPP-C phages had higher recruited numbers in waters above 75 m than in deeper waters, while MPP-B phages showed higher recruited numbers between 75 and 100 m, especially during summer and autumn (Fig. 4A). The time-averaged abundances indicated that the recruited numbers of MPP-C phages reached peak at 45 m and MPP-B phages peaked at 75 m (Fig. 4B). The MPP-C phages almost disappeared below 150 m, but MPP-B phages remained detectable between 150 and 250 m (Fig. 4B). The depth profile of MPP-B phages was consistent with a previous PCR-based study reporting that the MPP-B phages in the Red Sea were more abundant at depths [37]. Together, these results indicated that the MPP-C and MPP-B phages have distinct depth profiles in the oceans, with the MPP-C phages showing higher relative abundances in surface waters than in deep water and the MPP-B phages being relatively higher in deeper waters.

**Fig. 5:**
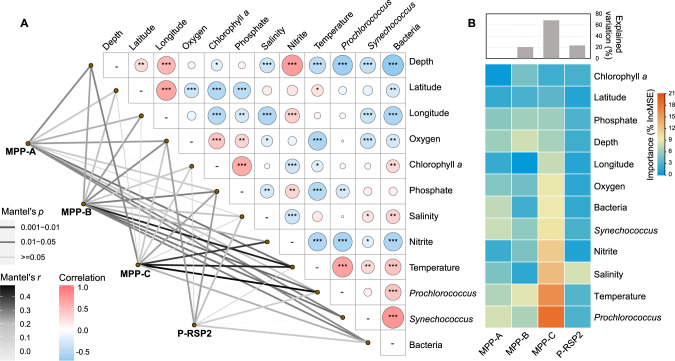
Environmental drivers for cyanopodovirus distribution patterns. **A**. The distribution of a cyanopodovirus clade was related to each environmental factor by Mantel’s test. Line colors corresponded to the Mantel’s *r* statistic for the distance correlations, and line widths denoted the statistical significance based on 999 permutations. Pairwise comparisons of environmental factors with a color gradient denoting Pearson’s correlation coefficient and *p* values were shown in the circles (*p < 0.05, **p < 0.01, ***p < 0.001). **B** Variable selection and estimating predictability based on random forest machine-learning algorithm. Histograms represented the percentage of explained variation. Heatmap showed the importance of different variables to the distribution of the four cyanopodovirus clades.

To assess how environmental variables shaped the global distribution of cyanopodovirus clades, we performed Mantel tests for the viromic datasets with available environmental parameters. The distribution of MPP-C phages was significantly correlated with location (latitude, longitude, depth), temperature, dissolved oxygen, chlorophyll *a*, nitrite, phosphate concentrations, and *Prochlorococcus* abundances (Fig. 5A). Among these parameters, the two most positively correlated ones were *Prochlorococcus* abundances (*p* < 0.01; *r* = 0.48) and temperature (*p* < 0.01; *r* = 0.45). In addition, the relative importance of environmental variables contributing to the distribution of cyanopodovirus clades was predicted by random forest modeling, which has shown high prediction accuracy in diverse ecological studies [68, 69]. *Prochlorococcus* abundances showed higher importance in predicting the distribution of MPP-C phages than in predicting other cyanopodovirus clades (Fig. 5B), indicating that the MPP-C phages could be an important predator of *Prochlorococcus*. Considering the high occurrence of MPP-C phages on a global scale, the tight coupling of MPP-C phages and *Prochlorococcus* emphasized the potential importance of MPP-C phages in the mortality and community structure of *Prochlorococcus* in the world’s oceans.

## Discussion

In this study, we reported the genomic analyses, infection kinetics, and biogeographic patterns of cyanopodoviruses in the MPP-C clade that we isolated from the South China Sea. The overall genomic architecture of the MPP-C phages resembled that of T7-like cyanopodoviruses in the MPP-A, MPP-B, and P-RSP2 clades. However, the MPP-C phages were the only cyanopodoviruses that do not encode DNA *pol* or AMGs, and their genomes were the smallest among sequenced cyanophages. Infecting *Prochlorococcus* MED4, the MPP-C phages showed much longer latent periods than MPP-B phages. By analyzing the global viromic datasets, we revealed that the MPP-C phages contributed to almost half of total cyanopodovirus reads across the global surface ocean. While the MPP-B phages showed higher relative abundances in DCM and Mix waters than in surface waters, the MPP-C phages were relatively more abundant in surface waters than in deep waters. With different depth profiles in the oceans, the MPP-B and MPP-C phages could affect the mortality of *Prochlorococcus* cells in a broad range of environmental conditions.

The lack of DNA *pol* suggested that the DNA replication mechanism of the MPP-C phages may be different from that of podoviruses containing DNA *pol*. The DNA replication complex (replisome) of coliphage T7 is made up of only four proteins: T7 DNA polymerase, T7 single-stranded DNA-binding protein, T7 helicase/primase bifunctional protein, and a processivity factor (thioredoxin) from the host *Escherichia coli* [70, 71]. While the MPP-A, MPP-B, and P-RSP2 phages all encode their own DNA polymerase and are likely to use similar DNA replication mechanisms as T7, we hypothesize that the MPP-C phages could replicate their genomes by one of three alternative mechanisms. First, the MPP-C phages may recruit the host DNA polymerase I, which belongs to the same DNA polymerase family as those of T7 and cyanopodoviruses. However, DNA polymerase I of prokaryotes is mainly involved in the repair of damaged DNA, but not genome replication [72]. It would be interesting to know whether the MPP-C phages can modify the host DNA polymerase I for viral genome replication. Second, the MPP-C phages may recruit the host DNA polymerase III, which consists of 10 unique subunits and is used by bacteria for genome replication [73]. To recruit host DNA polymerase III for viral DNA replication, *Bacillus subtilis* phage SPP1 encodes its own initiation protein and several other proteins to interact with the host DNA replication machinery to recognize the viral origin of replication, instead of the host one [74]. The MPP-C phages encode two proteins related to DNA replication (single-stranded DNA-binding protein and DNA helicase/primase bifunctional protein) (Fig. 1A), and thus these proteins may interact with the host DNA polymerase III. Third, we cannot rule out the possibility that the MPP-C phages may encode a DNA polymerase gene that is very divergent from currently known DNA polymerase genes even though no conserved domain corresponding to known DNA polymerase can be detected in the whole genomic sequences of MPP-C phages. Future studies on the DNA replication mechanism of the MPP-C phages will provide novel insights into viral DNA replication processes.

Due to its high conservation in previously sequenced podovirus genomes, DNA *pol* has been widely used as a genetic marker in quantification and diversity studies of marine cyanopodoviruses [7, 36, 37, 44–46]. However, our discovery of the MPP-C phages provided evidence that the T7-like DNA *pol* cannot fully assess all marine cyanopodoviruses. Thus, we suggest that the nine core genes shared by all marine cyanopodoviruses could serve as suitable genetic markers for marine cyanopodoviruses, especially the major capsid protein (Supplementary Figure 3). The suitable marker genes will help to more accurately measure distinct populations within the marine virioplankton and to quantify virus-mediated ecological processes. For example, in the North Pacific Subtropical Gyre, a polony method using DNA *pol* estimated that the abundance of cyanopodoviruses was 1.3–4.4 fold lower than that of cyanomyoviruses [45]. Given the high occurrence of MPP-C phages in the global ocean, our finding implies that the abundance of cyanopodoviruses could be much higher than previously thought. In addition, cyanopodovirus-mediated mortality of *Prochlorococcus* cells in the North Pacific Subtropical Gyre may be higher than estimated using DNA *pol* [75].

The distribution of viruses in the ocean is generally influenced by the availability of their hosts and the competitive ability of viruses under variable environmental factors [48]. We found that the MPP-C phages were relatively more abundant in surface waters than in deeper waters, while the MPP-B phages were relatively more abundant in deeper layers of the photic zone (Fig. 4B). Similar ecotype-specific depth profiles have been found in *Prochlorococcus* field populations, with the high-light-adapted ecotypes more abundant in surface waters and the low-light-adapted ecotypes in deeper waters [76]. In our study, the MPP-C phages were isolated from the surface waters using the high-light-adapted ecotype *Prochlorococcus* MED4 as the host. It remains to be tested whether the abundances of MPP-C and MPP-B phages are correlated to those of different *Prochlorococcus* ecotypes. Besides the availability of host cells, phages appear to have evolved different strategies to adapt to the environmental availability of resources. For example, the MPP-A, MPP-B, and P-RSP2 cyanopodoviruses encode at least two AMGs which could enable phages to tailor host metabolism and likely provide an advantage for phages to adapt to the environments [54, 77]. However, the MPP-C phages do not encode any of the six auxiliary metabolic genes (Fig. 1B). Two MPP-B phages (P-SSP7 and P-GSP1) isolated on the same host, which both carry AMGs involved in photosynthesis (*psbA* and *hli*), nucleotide synthesis (*rnr*) and carbon metabolism (*talC*) (Fig. 1), appear to have high relative abundances in both surface waters and deeper waters. While many factors could contribute to this phenomenon, we speculate that light intensity may play a role: host cells in surface waters can derive more energy from light than those in deep waters, thus providing more resources for the propagation of phage progeny, especially for MPP-C phages, which are small and need less resources (e.g., nucleotides). Meanwhile, the existence of AMGs in MPP-B phages is likely to benefit cyanophages at depths with low photosynthetic energy from host cells, enabling the ecological niche expansion of cyanophages in unfavorable conditions.

The biogeographic patterns of phages also depend on their life history traits, such as virion size, host range, infection kinetics, gene content, or lytic/lysogenic infection, etc. [38]. Compared to cyanopodoviruses that we measured in this study and from the literature, MPP-C phages differ in a number of properties, including smaller virions, smaller genome sizes, and longer latent periods. Previous studies suggested that smaller viruses could encounter host cells faster due to greater diffusivity in the marine environment, thus potentially increasing the probability of successful infection [78]. Although it remains to be confirmed whether the sizes of MPP-C phages are smaller than those of all the other cyanopodoviruses, we speculate that the small virion sizes may confer MPP-C phages a competitive advantage in the oligotrophic surface oceans where the abundance of the host is generally low and viral decay is high due to strong UV radiation. In summary, the depth profiles of MPP-C and MPP-B phages may be related to their distinct gene contents and infection kinetics. Similar to *Prochlorococcus* ecotypes [76], our study suggested that cyanopodoviruses may also evolve into different clades with distinct adaptation mechanisms to enable cyanophages to infect host cells in a broader range of environmental conditions.

## Materials and Methods

### Phage isolation and purification

The axenic host strain *Prochlorococcus* MED4 was grown in the Port Shelter (Hong Kong) seawater–based Pro99 [79] medium at 23 °C with continuous light of 25 µmol photons m^–2^ s^–1^. The surface seawater samples for phage isolation were collected in December 2015 at the Yongxing Harbor off the Yongxing Island, which is located in the South China Sea (16°50'14.14′)′ N, 112°19'34.30′′ E). The collected seawater samples were filtered through 0.22 μm pore-sized syringe filters and then kept in the dark at 4 °C. To isolate cyanophages infecting *Prochlorococcus* MED4, filtered seawater samples were mixed with *Prochlorococcus* MED4 in agarose plates with the helper strain *Alteromonas* sp. EZ55, following a previously described pour plating method [22]. Plates were incubated at 23 °C under continuous light (25 μmol photons m^–2^ s^–1^). Well isolated plaques were picked from the agarose plates, dissolved in the Pro99 medium, and purified with another two rounds of plating to form plaques.

### Determining the host ranges of isolated cyanophages

The host ranges of the newly isolated cyanopodoviruses were determined using spot assays [80]. The cyanopodoviruses were challenged against nine *Prochlorococcus* strains (MED4, MIT9301, MIT9312, MIT9313, AS9601, NATL1A, NATL2A, MIT9215, MIT9202) and three marine *Synechococcus* spp. strains (WH8012, WH7803, WH8102). Cyanobacterial cultures were plated on agarose plates together with *Alteromonas* sp. EZ55 as previously described [22]. After the agarose plates solidified, two microliters of each phage lysate were added to the surface of plates. After incubation under continuous light for about one to two weeks, the formation of clear plaques where lysates were added indicates successful phage infection of the cyanobacterial strain in the plate. Tests were repeated at least three times.

### Transmission electron microscopy (TEM)

The morphologies of three representative phages (P-SCSP1a, P-SCSP1u and P-SCSP2) were investigated with TEM. In brief, 3 μl of phage lysate were placed on formvar, carbon-coated copper electron microscopy grids and allowed to adsorb for 30 s. Phage particles were negatively stained with 2% (w/v) uranyl acetate for 30 s. Excess stain was removed with a filter paper and grids were air dried before examination with a Thermo-Fisher Talos L120C electron microscope hosted in the Biological Cryo-EM Center of the Hong Kong University of Science and Technology.

### Infection kinetics of cyanopodoviruses

Infection kinetics of phages from different cyanopodovirus groups were determined by one step growth experiments [81]. Freshly prepared cyanophage lysates were used to infect exponentially growing *Prochlorococcus* MED4 cultures (~10^7^ cells per ml) at a multiplicity of infection of 0.1 in triplicate at 21 °C under continuous light (10 µmol photons m^–2^ s^–1^). Infected cultures were taken every 2 hours after inoculation and filtered through 0.2 μm polycarbonate filters. Extracellular phage genomic DNA was quantified using quantitative PCR (qPCR), which provides ~1:1 ratio relative to intact phage numbers for both MPP-B and MPP-C phages (Supplementary Table 3). All qPCR assays were run in triplicate following our previous protocol [82]. Each qPCR amplification contained 4.6 μl template, 0.2 μl forward primer (10 μM), 0.2 μl reverse primer (10 μM), and 5 μl iTaq Universal SYBR Green Supermix. Primers were listed in Supplementary Table 4. Reactions were carried out on a LightCycler 480 Real-Time PCR System (Roche). The qPCR program contained one activation step of 95 °C for 5 min followed by 45 amplification cycles of 20 s denaturation at 95 °C and 60 s annealing and elongation step at 60 °C, and a melting curve analysis at the end.

### Genome sequencing and gene annotation

To obtain phage DNA for genome sequencing, cyanophage isolates were used to infect *Prochlorococcus* MED4 cultures to generate 250 ml phage lysates. The phage lysates were filtered through 0.22 μm filters and concentrated using a published FeCl_3_ flocculation method [83]. After resuspension, the phage concentrates were subjected to dialysis by the TM buffer (0.25 M sucrose, 0.05 M Tris, pH 7.4, 5 mM MgC1_2_). The phage concentrates were treated with 55 μg/ml proteinase K before DNA extraction using the Zymo Clean & Concentrator-5 kit. Viral genomic DNA was sheared into small fragments of around 300 bp in length using the Covaris S220 Focused-ultrasonicator. DNA sequencing libraries were prepared using the NEBNext Ultra II DNA library prep kit and sequenced using the HiSeq 4000 platform (Illumina). The sequencing data generated from each library contains around 2 Gb of 150 bp paired-end reads.

Raw reads were trimmed and filtered using Trimmomatic v0.36 to remove adaptor sequences and low-quality reads [84]. PRINSEQ v0.20.4 was employed to remove redundant sequences [85]. Clean reads were mapped against the *Prochlorococcus* MED4 genome to remove the host DNA fragments using Bowtie2 [86]. Remaining reads were normalized using bbnorm (https://jgi.doe.gov/data-and-tools/bbtools/) with a target coverage of 150 and assembled using the IDBA assembler v1.1.0 [87] with a maximum k-mer of 130 and a step size of 4. Prokka v1.13 [88] was used to predict and annotate genes in the assembled viral genomes with an e-value of 10^-5^ and coverage of 50% as the annotation threshold. The complete genome sequences of our newly isolated phages were submitted to the GenBank database (Table 1).

Pairwise average nucleotide identity (ANI) of cyanopodovirus genomes was performed using the pyani (v0.0.12) in ANIm mode (https://pypi.org/project/pyani/). Heatmap was plotted by R (v4.1.2). Core genes of cyanopodoviruses were identified by searching for the protein orthologs shared among the 18 newly sequenced cyanopodovirus isolates and the 21 known cyanopodoviruses using BLASTp and HMM search following previous methods [31]. Briefly, genes were considered orthologs if they were reciprocal best BLASTp hits (e-value ≤ 1e-5) and the alignment covered at least 75% of the length of the shorter gene. The HMM search was further used to identify divergent orthologues that may be missed by the BLAST-based search. The pan- and core-genome curves of the 39 cyanopodoviruses were generated using a random sampling algorithm and plotted as a function of the number of genomes analysed by R. The amino acid sequences of concatenated nine core genes and each individual core gene were used to construct phylogenetic trees to analyze the evolutionary relationships of cyanopodoviruses. Protein sequences were aligned by MUSCLE (v3.8.1551) [89], and phylogenetic trees were constructed with the maximum likelihood method using IQ-TREE (v2.0.3) with 1,000 ultrafast bootstraps [90, 91].

### Metagenomics analyses

To estimate the relative abundances and overall distributions of different marine cyanopodovirus clades, fragment recruitment analyses using marine virome data sets from the Tara Oceans expedition, the South China Sea, and the ALOHA station were carried out by BLAST with an e-value <10^-5^ as described previously [92, 93]. Previous studies have revealed that 95% ANI was a suitable threshold for recovering a set of closely related phage genomes [8, 10, 67]. Hence, in our study, 95% identity was further used to target reads identical to different cyanopodovirus clades across the global oceans. A coverage breadth >90% was set to recruit the mapped reads. If a read was recruited to more than one phage genome, the read was assigned to the phage with the highest bitscore. Recruitment data were normalized according to viral genome size and dataset size to estimate the kb recruited per kb of genome per Gb of metagenome (KPKG) as in previous studies [92, 93]. Phages with <40 % of covered genomes were regarded as absent and given a KPKG value of 0 [94, 95]. Samples with the highest KPKG value less than 0.001 for four clades were considered to have low number of cyanophages and omitted from our subsequent analyses to avoid bias. The global distributions and depth profiles of different cyanopodovirus clades were plotted using the ggplot2 package in R. The Mantel test was performed to assess the correlation between the recruitment frequency of cyanopodoviruses and environmental variables using the R package ‘vegan’. The contributions of different environmental variables in predicting the distribution of cyanopodoviruses were analyzed by random forest analysis using the R package RandomForest.

## Supplementary information

Supplementary material

Supplementary Table 1

Supplementary Table 2

## Data Availability

The complete genome sequences of our newly isolated phages are available on the GenBank database under the following accession numbers: OM416763–OM416780. Other Supplementary Information is available for download on the ISME website.
