# Supplementary material for: Abundant and cosmopolitan lineage of cyanopodoviruses lacking a DNA polymerase gene
Source: ISME J. 2022 Nov 10;17(2):252–62. doi: 10.1038/s41396-022-01340-6 (PMC9860041; doi:10.1038/s41396-022-01340-6)
Supplement: Supplementary file 1 — Supplementary material [file 41396_2022_1340_MOESM1_ESM.docx]

Supplementary Information for

**Abundant and cosmopolitan lineage of cyanopodoviruses lacking a DNA polymerase gene**

Lanlan Cai, Yue Chen, Shiwei Xiao, Riyue Liu, Maoqiu He, Rui Zhang, Qinglu Zeng

To whom correspondence may be addressed. Email: zeng@ust.hk

**This file includes:**

Supplementary Figures 1 to 3

Supplementary Tables 3 to 4

**
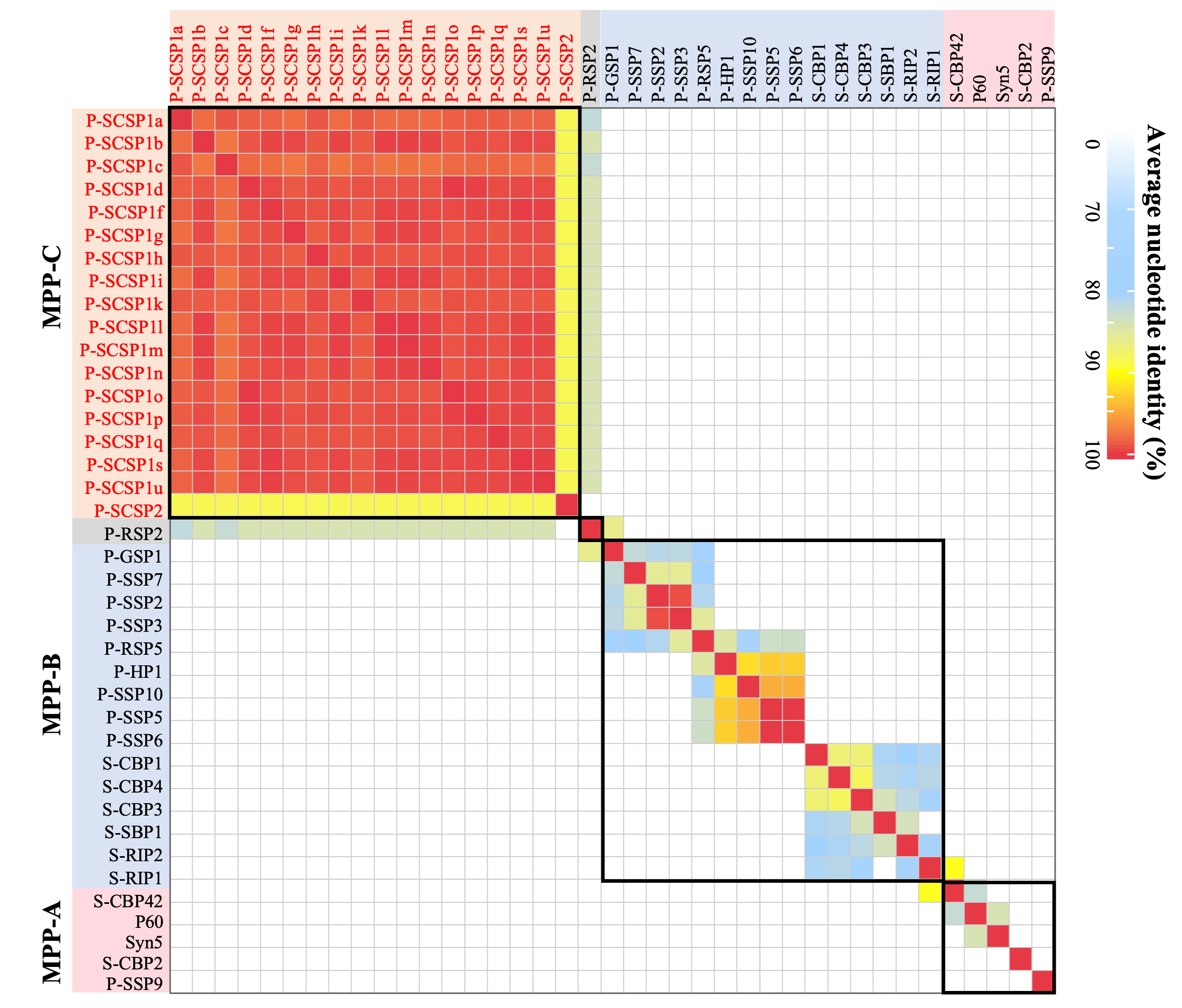
**

**Supplementary Figure 1. Pairwise genome comparison between different cyanopodoviruses.**

The names of cyanophages belonging to different clades were shaded in different colors: pink for MPP-A, blue for MPP-B, orange for MPP-C, and gray for P-RSP2. Phages isolated in this study were indicated in red. Color bar on the right showed the values of average nucleotide identity between two cyanophage genomes.


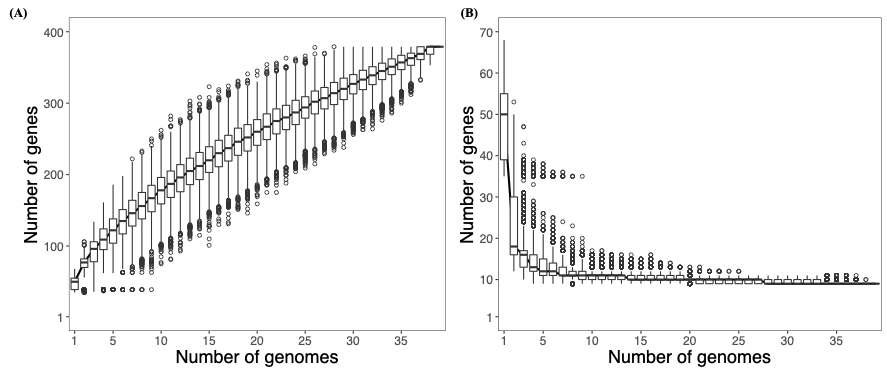


**Supplementary Figure 2. Pan- and core-genomes of cyanopodoviruses.**

The gene numbers of pan-genome (**A**) and core-genome (**B**) were plotted as a function of the number of analyzed genomes from the 18 newly isolated and 21 previously available cyanopodoviruses. The line connecting the box plots showed the median of the gene numbers. For each box plot, the bottom and top of the box represented the first and third quartiles, respectively. The ends of whiskers showed the upper and lower extremes, and circles indicated outliers.


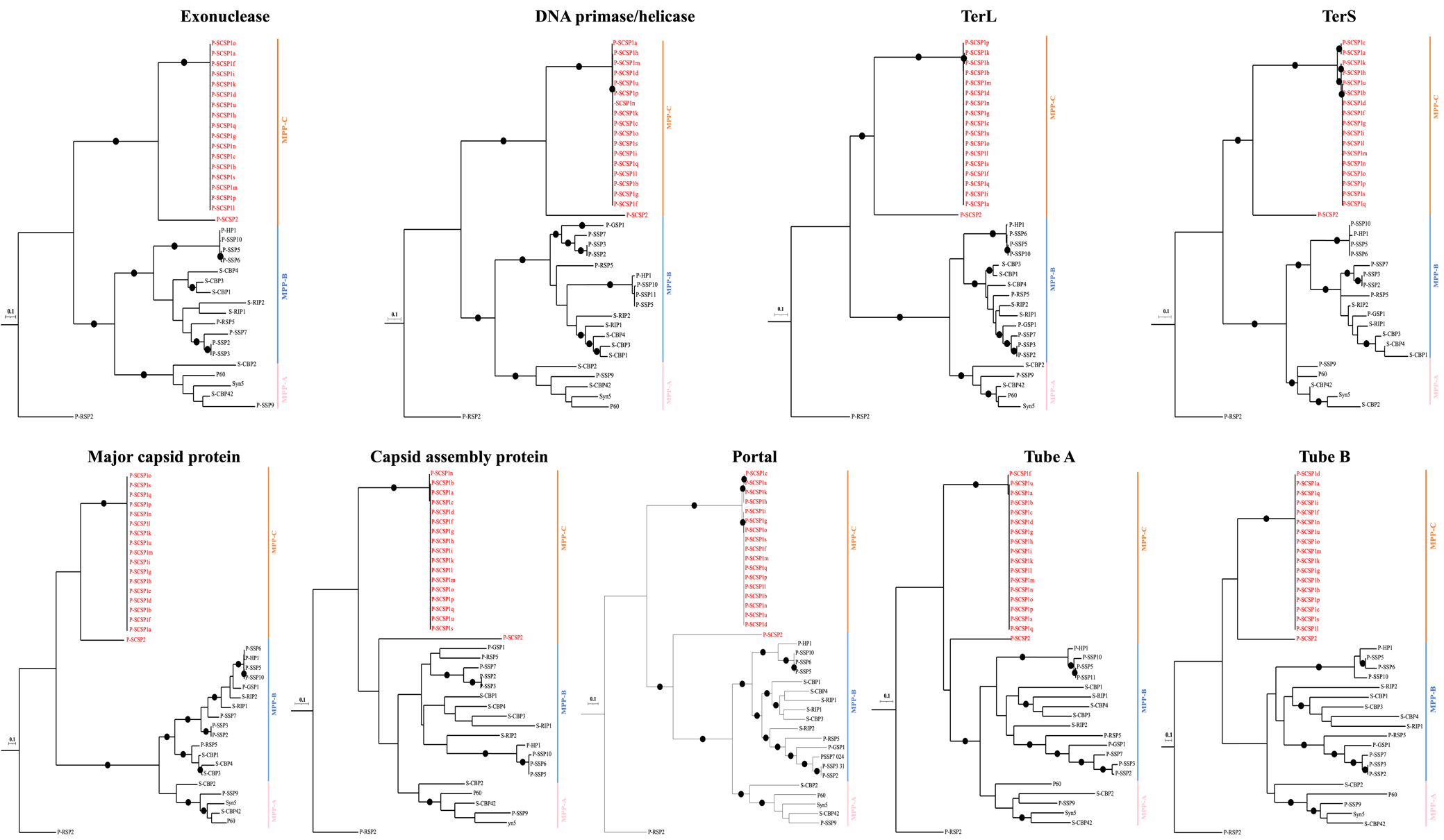


**Supplementary Figure 3. Phylogenetic trees built using the amino acid sequences of the nine core genes shared among cyanopodoviruses.**

Black dots indicated bootstrap values larger than 80%. The names of phages isolated in this study were highlighted in red.

**Supplementary Table 3. Comparison of phage count between qPCR and epifluorescence microscopy (EFM).**

| Phage | Clade | EFM/qPCR |
| --- | --- | --- |
| P-SCSP1a | MPP-C | 0.91 |
| P-SCSP1b | MPP-C | 0.85 |
| P-SCSP1c | MPP-C | 0.93 |
| P-SCSP1g | MPP-C | 0.92 |
| P-SCSP1h | MPP-C | 0.88 |
| P-SCSP1l | MPP-C | 0.85 |
| P-SCSP1m | MPP-C | 0.93 |
| P-SCSP1s | MPP-C | 0.84 |
| P-SCSP2 | MPP-C | 0.93 |
| P-GSP1 | MPP-B | 0.91 |
| P-SSP7 | MPP-B | 0.87 |

The number of viral genomic DNA was determined by qPCR (see Materials and Methods). The number of intact phage particles after DNase I treatment was determined by EFM. The results showed a good correlation of phage counts between qPCR and EFM for both MPP-B and MPP-C phages. For quantification of intact phages by EFM, phage lysate was treated with 1 µg/mL DNase I at room temperature for 1 hour and then filtered onto 0.02 μm pore-size membrane filters (Anodisc 25, Whatman). The filters were stained with SYBR Green I (Invitrogen) for 15 minutes and observed in a fluorescence microscope (Nikon Eclipse Ts2R, Japan). Phage particles were manually counted from at least 10 microscopic fields, ensuring a minimum count of 500 for each sample.

| **Supplementary Table 4. Quantitative PCR primers used to measure phage DNA copy numbers.** | | | |  |
| --- | --- | --- | --- | --- |
| **﻿Cyanophage** | **Target gene** | **﻿Forward primer (5’ to 3’)** | **﻿Reverse primer (5’ to 3’)** | **﻿References** |
| P-SCSP1 subgroup | Portal | CAGGCAGCTGGTAAATCACAA | TCTCCTGGTCTTTGTATCGCT | This study |
| P-SCSP2 | Portal | TGGAAGGTTCTACAGCAGCA | CCACGACTACCAACAGCTTG | This study |
| P-SSP7 | *DNA pol* | AAACACTTCCGCCCTTACCT | CTGCAACGAAAGGGAATTGT | Lindell *et al*. 2007 |
| P-GSP1 | *DNA pol* | GATTAGAGCACCGAGTCGC | TCGTTTAGGAGTGAACAACGC | This study |
|  |  |  |  |  |
| **Reference** |  |  |  |  |
| ﻿Lindell D, Jaffe JD, Coleman ML, Futschik ME, Axmann IM, Rector T, et al. Genome-wide expression dynamics of a marine virus and host reveal features of co-evolution. Nature. 2007;449(7158):83-86. | | | | |
